# Suitability of the animated activity questionnaire for use as computer adaptive test: establishing the AAQ-CAT

**DOI:** 10.1007/s11136-023-03402-4

**Published:** 2023-04-03

**Authors:** Gregor Liegl, Leo D. Roorda, Caroline B. Terwee, Martijn Steultjens, Ewa M. Roos, Francis Guillemin, Maria Grazia Benedetti, Hanne Dagfinrud, Alessandra de Carvalho Bastone, Wilfred F. Peter

**Affiliations:** 1 Center for Patient-Centered Outcomes Research, Charité – Universitätsmedizin Berlin, corporate member of Freie Universität Berlin and Humboldt-Universität zu Berlin, Berlin, Germany; 2 Amsterdam Rehabilitation Research Center | Reade, Amsterdam, The Netherlands; 3 Department of Epidemiology and Data Science, Amsterdam UMC, Vrije Universiteit Amsterdam, Amsterdam, The Netherlands; 4 Amsterdam Public Health Research Institute, Amsterdam, The Netherlands; 5 School of Health and Life Sciences, Glasgow Caledonian University, Glasgow, UK; 6 Institute of Sports Science and Clinical Biomechanics, University of Southern Denmark, Odense, Denmark; 7 EA 4360 APEMAC, Inserm CIC-EC 1433, University Hospital, Université de Lorraine, Nancy, France; 8 Physical Medicine and Rehabilitation Unit, Istituto Ortopedico Rizzoli, Bologna, Italy; 9 Diakonhjemmet Hospital, Oslo, Norway; 10 University of Oslo, Oslo, Norway; 11 Department of Physical Therapy, Universidade Federal dos Vales do Jequitinhonha e Mucuri (UFVJM), Diamantina, MG Brazil

**Keywords:** Hip and knee osteoarthritis, Patient reported outcomes, Physical function, Performance outcomes, Item-response theory, Computer-adaptive testing

## Abstract

**Purpose:**

The animated activity questionnaire (AAQ) is a computer-based measure of activity limitations. To answer a question, patients choose the animation of a person performing an activity that matches their own level of limitation. The AAQ has not yet been tested for suitability to be applied as computer-adaptive test (CAT). Thus, the objective of this study was to develop and evaluate an AAQ-based CAT to facilitate the application of the AAQ in daily clinical care.

**Methods:**

Patients (*n* = 1408) with hip/knee osteoarthritis from Brazil, Denmark, France, The Netherlands, Norway, Spain, and the UK responded to all 17 AAQ items. Assumptions of item-response theory (IRT) modelling were investigated. To establish item parameters for the CAT, a graded response model was estimated. To evaluate the performance of post-hoc simulated AAQ-based CATs, precision, test length, and construct validity (correlations with well-established measures of activity limitations) were evaluated.

**Results:**

Unidimensionality (CFI = 0.95), measurement invariance (*R*^2^-change < 2%), and IRT item fit (S-X^2^ *p* > .003) of the AAQ were supported. Performing simulated CATs, the mean test length was more than halved (≤ 8 items), while the range of precise measurement (standard error ≤ 0.3) was comparable to the full AAQ. The correlations between original AAQ scores and three AAQ-CAT versions were ≥ 0.95. Correlations of AAQ-CAT scores with patient-reported and performance measures of activity limitations were ≥ 0.60.

**Conclusion:**

The almost non-verbal AAQ-CAT is an innovative and efficient tool in patients with hip/knee osteoarthritis from various countries, measuring activity limitations with lower respondent burden, but similar precision and construct validity compared to the full AAQ.

**Supplementary Information:**

The online version contains supplementary material available at 10.1007/s11136-023-03402-4.

## Introduction

Osteoarthritis is a highly prevalent chronic disease and a major cause of activity limitations in affected patients [1, 2]. With hip and knee being two of the most affected joints [1], patients are often particularly limited in lower body functions and associated activities of daily living (ADL). Consequently, limitations in performing physical activities is an important outcome in the field of hip and knee osteoarthritis [3].

By now, many different measures of activity limitations have been developed [4], with patient-reported outcome (PRO) and performance-based outcome (PerfO) measures being the most frequently used assessment types [4, 5]. In PRO measures, respondents rate their perceived level of activity limitations by responding to self-report items in a questionnaire, while PerfO measures assess a patient’s performance of physical tasks in a standardized test environment [4]. PRO measures are easy to use and cheap but subjective with regard to the interpretation of the terms used in the questionnaire (i.e., ‘difficulty’) and to the adopted reference frame of the respondent (i.e., the situation or status the subject relates) [6]. It has been shown that PRO measures are more influenced by subjective patient variables than PerfO assessments [6–9]. In contrast, PerfO measures lead to more objective assessments, but are more resource-intense and burdensome to the patients [10]. Moreover, while PRO measures allow to capture a broad range of different activities, PerfO measures usually focus on a very specific activity and are often used as single-task measures [11]. In sum, several previous research findings indicate that PRO and PerfO measures may assess related but yet different constructs, and that respective results should only be compared with caution [12, 13].

To combine the advantages of PRO and PerfO measures within one instrument, the animated activity questionnaire (AAQ) was developed [14, 15]. The AAQ is an online-based measure of activity limitations for patients with hip and knee osteoarthritis. Each of its 17 items consists of several videos of an animated avatar performing a specific ADL task. To answer an item, patients choose the animation that best matches their own activity limitation level. Resource-intensity of the AAQ is comparable to computer-based PRO measures. At the same time, by showing animations of activities in a standardized real life situation and environment, the influence of the patient’s reference frame is expected to be minimized [14, 16]. Moreover, the AAQ is almost non-verbal, potentially reducing validity problems due to differences in literacy across patients, and allows for cross-language application with little translational efforts [16, 17]. Thus, the AAQ is being discussed as a suitable alternative for PerfO measures in largescale studies [14].

Several studies indicated good psychometric characteristics of the AAQ [14, 16–19]. However, item response theory (IRT) methods, allowing for validating an instrument on item-level [20], has not yet been applied to psychometrically evaluate the AAQ. Using IRT, ability estimates can be assessed on an interval scale and statistical precision and power can be improved [20]. Estimating an IRT model provides individual parameters for each item of a measure [21–23]. A major advantage of using IRT item parameters for scoring is that item sets can be optimized by administering only the most relevant and precise items for a given ability level. One method of item set optimization is the application of computerized adaptive tests (CAT) [23]. In a CAT, a computer algorithm automatically selects the most informative items for an individual respondent, based on her or his answers given on previous items [23]. These algorithms are based on item parameters reflecting the individual statistical relationship between the latent construct of a measure and the responses to a given item. The application of CATs usually leads to a significant reduction in the number of items to be answered and in the time required to complete a questionnaire [21, 23, 24]. This seems to be particularly useful even for relatively short instruments, as study participants often have to complete not only a single questionnaire, but entire batteries of questionnaires, which can be very burdensome for patients and resource-intensive for those conducting these studies [24]. Moreover, to answer an AAQ item, patients must watch multiple videos simultaneously, which requires a certain level of concentration and attention. Therefore, administration as a CAT could lead to a significant reduction in patient burden.

Considering its computer-based nature, the AAQ seems to be very well-suited for being used as a CAT. Thus, the aims of this study were (1) to investigate if the items of the AAQ fulfill psychometric criteria for IRT modelling and (2) to establish IRT item parameters which can be used for the application as CAT. Moreover, (3) the performance of different CAT versions in terms of test length, precision, and construct validity will be evaluated based on post-hoc simulations.

## Methods

### Measures

#### Animated activity questionnaire (AAQ)

The AAQ is an online animated questionnaire containing 17 items of single ADL tasks [14, 15]. The development and selection of items was based on conceptual and theoretical considerations as well as focus groups with patients [14]. Each item simultaneously shows 3 to 5 animated videos of an avatar performing an ADL task. Patients select the animation that best matches their performance of the task in the past week, or “Not possible” (http://www.kmin-vumc.nl/_16_0.html). Responses are scored from 1 to 4/5/6 (depending on the number of response options), with higher response categories indicating more activity limitations. The AAQ is currently available in eleven languages at https://animatedactivityquestionnaire.com/. The AAQ showed high test–retest reliability (intraclass correlation = 0.97) and internal consistency (Cronbach’s alpha = 0.95) [14], next to other satisfactory psychometric properties with regard to responsiveness [19], construct validity, and cross-cultural validity [17]. AAQ scores are transformed to a 0–100 metric with higher scores indicating less activity limitations.

#### H/KOOS ADL subscale and PerfO measures

To investigate construct validity of the IRT-calibrated AAQ measure and related CAT scores, a disease-specific PRO and three PerfO measures were administered. As PRO measure, the ADL subscale of the hip disability and osteoarthritis outcome score (HOOS) [25] or knee injury and osteoarthritis outcome score (KOOS) [26] was used. The ADL subscales of the HOOS and KOOS are identical, therefore the same scale was used for both hip and knee patients (H/KOOS). The H/KOOS contains 17 questions about perceived difficulty in executing ADL tasks in the past week due to hip or knee problems, on a 5-point scale. A total H/KOOS score was calculated and transformed into a score ranging from 0 to 100, with higher scores indicating less activity limitation. In addition, three single-item PerfO measures were executed by a subsample of participants: the Stair Climbing Test (SCT; *n* = 324) [27], and the Timed Up and Go test (TUG; *n* = 396) [28], both measuring the time in which the activity is performed, and the 30 s Chair Stands Test (CST; *n* = 325) [29], which takes the number of sit to stands that was performed within 30 s. These measures were chosen from the most feasible, reliable and responsive measures recommended by OsteoArthritis Research Society International [30].

### Participants and data collection

The present study used data from various research projects on the development, translation and evaluation of the AAQ, which were collected between 2013 and 2019 [14, 16–19, 31]. The largest of these projects with 1239 participants was conducted to establish cross-cultural validity of the AAQ in 7 European countries, namely Denmark, France, Italy, the Netherlands, Norway, Spain, and United Kingdom [17]. However, Italian AAQ data (*n* = 203) were not considered for the present study because problems with cross-cultural validity have been identified [17]. In addition to the data mentioned above, Brazilian data were analysed that were collected as part of another AAQ validation project [31]. In all participating countries, patients aged over 18 years with a diagnosis of hip and/or knee OA according the ACR criteria [32] were invited to participate in the study, either by phone or when they visited the clinic where they receive treatment. If they agreed to participate, an information leaflet, an informed consent form, and a pre-stamped, pre-addressed envelope were sent or given to them personally. A consecutive sample of patients was recruited from different health care settings such as primary care, in-patient rehabilitation, and hospitals. The participants were sent a link to the online questionnaire. They completed the AAQ and the H/KOOS ADL subscale in consecutive order. A random subgroup was invited to visit the outpatient clinic to execute three performance-based tests after the AAQ and the H/KOOS were completed.

### Statistical analysis

To describe the characteristics of the study sample, descriptive statistics were used (see Table 1). Psychometric analyses were conducted following the analysis plan of the Patient-Reported Outcomes Measurement Information System (PROMIS) [33]. Table 2 provides an overview of psychometric properties and related research questions that were investigated, including analyses and statistics, criteria, and applied software.

Before IRT-based AAQ item parameters were estimated, assumptions of IRT modelling were checked [34]. We conducted a confirmatory factor analysis (CFA) of a one-factor model with a weighted least squares means and variance adjusted (WLSMV) estimator, which is a robust (scaled) variant of the diagonally weighted least squares estimator [33, 35]. Unidimensional model fit was evaluated by calculating the comparative fit index (CFI), the Tucker–Lewis index (TLI), the root mean square error of approximation (RMSEA), and the standardized root mean square residual (SRMSR) [33]. Since strictly unidimensional models have been discussed as too restrictive when applied to patient-reported data, the explained common variance (ECV) as well as Omega *H,* resulting from an exploratory bifactor model with one general and three specific group factors, were additionally used to evaluate ‘sufficient’ unidimensionality [33, 36, 37]. Residual correlations were calculated for each pair of items to investigate locale independence. Low residual correlations indicate that all covariation between items is explained by the common factor. Monotonicity, meaning that subjects with more severe activity limitation are more likely to score higher on each AAQ item, was evaluated using Mokken scale analysis [38]. Loevinger’s homogeneity coefficient *H* was used as an indicator of scalability for the total AAQ scale. For determining the discriminative power of each AAQ item, item specific *H*_*j*_ values were calculated [38]. Differential item functioning (DIF) analysis was used to examine measurement invariance across patient groups regarding age, gender, and country. With regard to DIF by country, we compared Brazilin data versus all other countries, because measurement invariance across the other 6 countries was demonstrated before [17]. To identify DIF, ordinal logistic regression was applied [39].

Since we did not assume all items to have equal discrimination based on the results of a previous study [14], a two-parameter IRT model, namely the graded response model (GRM), was fitted to estimate item parameters [40]. In GRMs, one slope and several (number of response options minus 1) threshold parameters are estimated for each item. While the slope parameter (a) specifies how strong an item is associated with the latent trait (discrimination), threshold parameters (*b*_*j*_) define the locations on the latent trait continuum at which item responses are most informative. Data from all countries were modelled together. Item fit was evaluated using the S-X^2^ statistic, assessing the discrepancy between observed and model-predicted item responses [41]. Item characteristic curves were checked for disordered thresholds [42].

To investigate the performance of applying the AAQ items as CAT, post-hoc simulations were conducted [43], based on the responses of the participants to the full set of AAQ items and the established GRM item parameters. The maximum Fischer Information (MFI) method was used to automatically select the most informative items [43]. For estimating scores indicating the individual level of activity limitations (theta), expected a posteriori (EAP) estimation was applied [43].

IRT-based AAQ scores (theta) were initially calibrated to the total sample mean of 0 and a standard deviation of 1, with higher scores indicating more activity limitations. In a second step, to enable comparisons with the original AAQ measure, expected sum scores were calculated based on the fitted IRT model [44] and subsequently transformed to the original 0–100 metric, with higher scores indicating less activity limitations.

The performance of three versions of the AAQ-CAT was compared to the full AAQ measure. The three CAT versions differed in the pre-specified stopping rule:

1. ‘CAT-17’: Administration of further items stops when the standard error becomes ≤ 0.3, with a maximum of all 17 AAQ items.
2. ‘CAT-10’: Administration of further items stops when the standard error becomes ≤ 0.3, with a maximum of 10 items.
3. ‘CAT-5’: Administration of further items stops when the standard error becomes ≤ 0.3, with a maximum of 5 items.

The root mean square error (RMSE) and the mean difference to original AAQ scores (bias) were calculated for the IRT-calibrated AAQ measure and the CAT versions. Mean test lengths (i.e., number of items administered) and precision (i.e., standard error) in dependence of a given activity limitation level was calculated for the different CAT versions and inspected graphically.

To investigate construct validity, AAQ scores were correlated with the H/KOOS ADL, the SCT, the TUG, and the CST. We hypothesized that correlation coefficients would be ≥ 0.60 [17].

For statistical analyses, R 3.6.2 was applied and the R packages catR, lavaan, lordif, mirt, mokken, and psych were used [35, 43–48].

## Results

### Sample characteristics

AAQ data from 1408 patients with hip/knee osteoarthritis from Brazil (*n* = 200), Denmark (*n* = 201), France (*n* = 190), The Netherlands (*n* = 425), Norway (*n* = 91), Spain (*n* = 99), and the UK (202) were included. Further demographic and clinical characteristics of the study sample regarding gender, age, body mass index (BMI), affected joint(s) and joint replacement are presented in Table 1.

**Table 1:** Sample characteristics (*n* = 1408)

Variables	
Female; *n* (%)	1037 (73.7)
Mean age (SD)	64.5 (9.6)
Mean BMI (SD)	28.6 (5.4)
Joint affected; *n* (%)	
Knee(s) only	854 (60.7)
Hip(s) only	287 (20.4)
Both	267 (19.0)
Total joint replacement; *n* (%)	
None	1008 (71.6)
Knee(s) only	209 (14.8)
Hip(s) only	159 (11.3)
Both	32 (2.3)
Country; *n* (%)	
Brazil	200 (14.2)
Denmark	201 (14.3)
France	190 (13.5)
Netherlands	425 (30.2)
Norway	91 (6.5)
Spain	99 (7.0)
United Kingdom	202 (14.3)
Measures of ability limitations; mean (SD)	
AAQ (0–100)	78.3 (17.3)
H/KOOS ADL (0–100)	64.6 (20.7)
Stair climbing test (SCT; s)	17.1 (9.9)
Timed up and go (TUG; s)	12.1 (7.1)
Chair stands test (CST; counts)	9.4 (6.8)

*AAQ* animated activity questionnaire, *BMI* body mass index, *H/KOOS ADL* ADL subscale of the Knee disability and Osteoarthritis Outcome Score (HOOS) or Knee injury and Osteoarthritis Outcome Score (KOOS), *n* sample size, *SD* standard deviation

### Psychometric properties

#### Criteria for IRT modelling

Results with regard to IRT-model assumptions are presented in Table 2. While results of the CFA were somewhat contradictory, bifactor analysis supported sufficient unidimensionality of the AAQ for IRT analysis. Residual correlations were lower than 0.2 in 97% of item pairs, supporting local independence and, thereby, indicating that one common factor explains almost all covariation across items. Monotonicity and IRT model fit were supported for all AAQ items. DIF analysis confirmed measurement invariance regarding age, gender, and country.

**Table 2:** Psychometric properties of the AAQ 17-items instrument

Psychometric properties and related research questions	Statistics/indices	Criterion	Software	Results
**Unidimensionality: Do all items of the measure assess a common construct?**				
Confirmatory factor analysis (CFA)^a^			lavaan (R package)	
	CFI	> 0.95		0.95
	TLI	> 0.95		0.94
	RMSEA	< 0.06		0.15
	SRMSR	< 0.08		0.08
Exploratory bifactor analysis			psych (R package)	
	ECV	> 0.70		0.76
	Omega *H*	> 0.80		0.84
**Local independence: Do the items relate only to the construct being measured?**				
Residual correlation matrix resulting from CFA	Residual correlations of item pairs (*r*_Res_)	$\le$ 0.20	lavaan (R package)	*r*_Res_ ≤ 0.20 in 97% of item pairs
**Monotonicity: Do the probabilities of affirmative responses to the items increase with increasing levels of the construct?**				
Mokken scale analysis	Scalability coefficient of the total scale (*H*)	> 0.50	mokken (R package)	0.60
**Measurement invariance: Is it valid to use the same IRT-model to compare these groups?**				
Differential item functioning by age (median split)	McFadden’s pseudo *R*^2^-change	< 2%	lordif (R package)	*R*^2^-change < 2% in 100% of items
Differential item functioning by gender (female versus male)				*R*^2^-change < 2% in 100% of items
Differential item functioning by country (Brazilian data versus data from all other countries^b^)				*R*^2^-change < 2% in 100% of items
**IRT model fit: Can the relationship between the items adequately be described by a GRM?**				
GRM fit	S-X^2^ *p*-value^c^	≥ 0.003	mirt (R package)	*p* ≥ 0.003 in 100% of items

*CFI* comparative fit index, *ECV* explained common variance, *IRT* item response theory, *GRM* graded response model, *H* Loevinger’s Homogeneity coefficient, *r* correlation coefficient, *RMSEA* root mean square error of approximation, *SRMSR* standardized root mean square residual, *TLI* Tucker-Lewis index

^a^Fit statistics are based on a weighted least squares means and variance adjusted (WLSMV) estimator, which is a robust variant of the diagonally weighted least squares estimator

^b^Measurement invariance across all included countries except Brazil has already been demonstrated (Peter et al. [17])

^c^S-X^2^ item fit statistics were evaluated after adjusting for multiple testing (*p* ≥ 0.003)

#### Item characteristics

As IRT assumptions were fulfilled, legitimating IRT modelling, a GRM was estimated for all 17 AAQ items. Item parameters as well as detailed characteristics regarding scalability and fit statistics are presented in Table 3. While slopes of most items were (close to) a = 2 or above, slopes for item 16 (‘putting on shoes’) and item 17 (’taking off shoes’) were considerably lower (a = 1.40 and a = 1.29, respectively). Threshold parameters ranged from − 1.56 (b1 of item 16; ‘putting on shoes’) to 3.69 (b4 of item 11; ‘sitting down on a chair’). Disordered thresholds were detected in four items (see Online Appendix Fig. A1). After collapsing the affected response categories and reanalyzing the data using a GRM, the newly estimated theta values matched the theta values of the original model (Pearson’s *r* = 1.00), indicating that the recoding of the items had no effect. Since a satisfactory fit of the items was found for the original model, we decided to retain the original response categories for all items. A test information plot is provided in Online Appendix Fig. A2, indicating highly reliable measurements (defined as marginal reliability ≥ 0.9 ≈ test information ≥ 10) between theta = -1 and theta = 4.

**Table 3:** AAQ item characteristics

Item		Monoto-nicity	GRM fit	GRM item parameters^a^					
Item ID	Item description	*H*_*i*_	S-X^2^ *p*-value^b^	a	b1	b2	b3	b4	b5
AAQ_01	Ascending stairs	0.613	0.369	2.743	0.126	0.960	1.954	2.798	–
AAQ_02	Descending stairs	0.580	0.232	2.386	− 0.191	0.698	1.802	2.275	3.096
AAQ_03	Walking outside on a flat surface	0.628	0.030	2.857	0.130	1.168	1.443	2.193	3.259
AAQ_04	Walking outside on uneven terrain	0.634	0.667	2.964	0.156	1.079	1.429	2.444	–
AAQ_05	Walking inside: starting walking after at least 15 min sitting	0.629	0.069	2.467	− 0.526	0.879	2.173	3.363	–
AAQ_06	Ascending a bridge	0.657	0.499	3.453	0.087	1.326	2.123	2.928	–
AAQ_07	Descending a bridge	0.648	0.035	3.132	0.005	1.406	2.221	3.092	–
AAQ_08	Picking up an object from floor	0.584	0.030	2.260	0.092	1.033	1.710	2.869	–
AAQ_09	Rising from the floor	0.560	0.073	1.983	− 0.240	0.987	1.859	–	–
AAQ_10	Rising from a chair	0.630	0.284	2.693	− 0.346	1.230	2.024	3.526	–
AAQ_11	Sitting down on a chair	0.620	0.480	2.734	− 0.105	1.312	1.823	3.690	–
AAQ_12	Rising from a sofa	0.669	0.158	2.789	− 0.886	0.590	1.553	2.629	–
AAQ_13	Sitting down on a sofa	0.633	0.012	2.779	− 0.400	0.898	1.606	2.800	–
AAQ_14	Rising from a toilet	0.633	0.385	2.964	0.072	0.929	2.002	3.195	–
AAQ_15	Sitting down on a toilet	0.622	0.751	2.727	0.186	1.126	2.234	3.430	–
AAQ_16	Putting on shoes	0.499	0.654	1.403	− 1.561	− 0.259	1.433	3.055	–
AAQ_17	Taking off shoes	0.461	0.008	1.289	− 0.461	0.505	3.144	–	–

*H*_*i*_ Loevinger’s Homogeneity coefficient (on item level), *GRM* graded response model

^a^In GRMs, one slope and several (number of response options minus 1) threshold parameters are estimated for each item. While the slope parameter (a) specifies how strong an item is associated with the latent trait, threshold parameters (b_j_) define the locations on the latent trait continuum at which an item responses are most informative

^b^S-X^2^ item fit statistics were evaluated after adjusting for multiple testing (*p* < 0.003)

#### Performance of the AAQ CAT

Results of the IRT-calibrated AAQ measure and each AAQ-CAT version regarding measurement characteristics and construct validity in comparison to original AAQ scores are summarized in Table 4. Scores derived from the IRT-calibrated AAQ measure as well as score estimates from all CAT versions were very close to original AAQ scores, with Pearson correlations of *r* ≥ 0.95. RMSE and bias were comparable between the different CAT versions.

**Table 4:** Comparison of measurement characteristics and construct validity between IRT-based AAQ scores and different CAT versions with original AAQ scores

	Original AAQ score	IRT-based AAQ score	CAT-17	CAT-10	CAT-5
Mean test length	17 items	17 items	8.0 items	6.6 items	4.8 items
AAQ score mean (SD)	78.3 (17.3)	78.5 (16.8)	78.4 (16.7)	78.4 (16.7)	78.4 (16.6)
AAQ score range	0.0–100.0	2.3–98.6	8.0–98.6	8.0–98.6	8.0–98.6
RMSE	–	1.71	5.02	5.05	5.39
Bias (mean difference to original AAQ)	–	0.17	0.11	0.13	0.16
Pearson correlation with full AAQ (95% CI)	–	1.00 [1.00, 1.00]	0.96 [0.95, 0.96]	0.96 [0.95, 0.96]	0.95 [0.95, 0.96]
Pearson correlation H/KOOS ADL (95% CI)	0.73 [0.71, 0.76]	0.73 [0.70, 0.75]	0.68 [0.65, 0.71]	0.68 [0.65, 0.71]	0.68 [0.65, 0.71]
Spearman correlation with SCT (95% CI)	− 0.68 [− 0.74, − 0.62]	− 0.68 [− 0.74, − 0.62]	− 0.66 [− 0.72, − 0.58]	− 0.66 [− 0.72, − 0.59]	− 0.66 [− 0.72, − 0.59]
Spearman correlation with TUG (95% CI)	− 0.61 [− 0.68, − 0.54]	− 0.62 [− 0.68, − 0.54]	− 0.60 [− 0.66, − 0.52]	− 0.60 [− 0.66, − 0.52]	− 0.60 [− 0.67, − 0.52]
Spearman correlation with CST (95% CI)	0.60 [0.52, 0.67]	0.60 [0.52, 0.67]	0.60 [0.52, 0.67]	0.60 [0.52, 0.67]	0.60 [0.52, 0.67]

*AAQ* animated activity questionnaire, *CAT* computer adaptive test, *CI* confidence interval, *CST* chair stands test, *H/KOOS ADL* ADL subscale of the knee disability and osteoarthritis outcome score (HOOS) or knee injury and osteoarthritis outcome score (KOOS), *RMSE* root mean square error, *SCT* stair climbing test, *SD* standard deviation, *TUG* timed up and go test

Items 4, 6, 7, 12, 13, and 14 had the highest exposure rates in each CAT version, indicating that these items are the most informative items for the sample. This is in line with Table 3, showing that all of these items had comparatively high slopes.

**Fig. 1:**
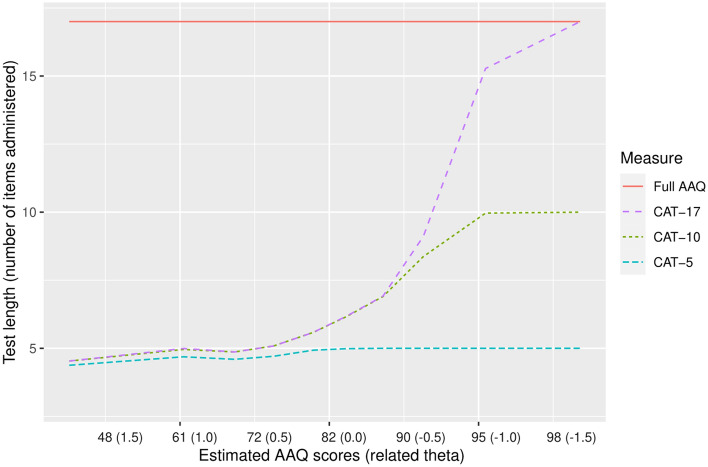
Conditional test length: average number of items administered (*y*-axis) for estimated AAQ score and related theta deciles (*x*-axis) in the study sample. Higher AAQ scores indicate less activity limitations

**Fig. 2:**
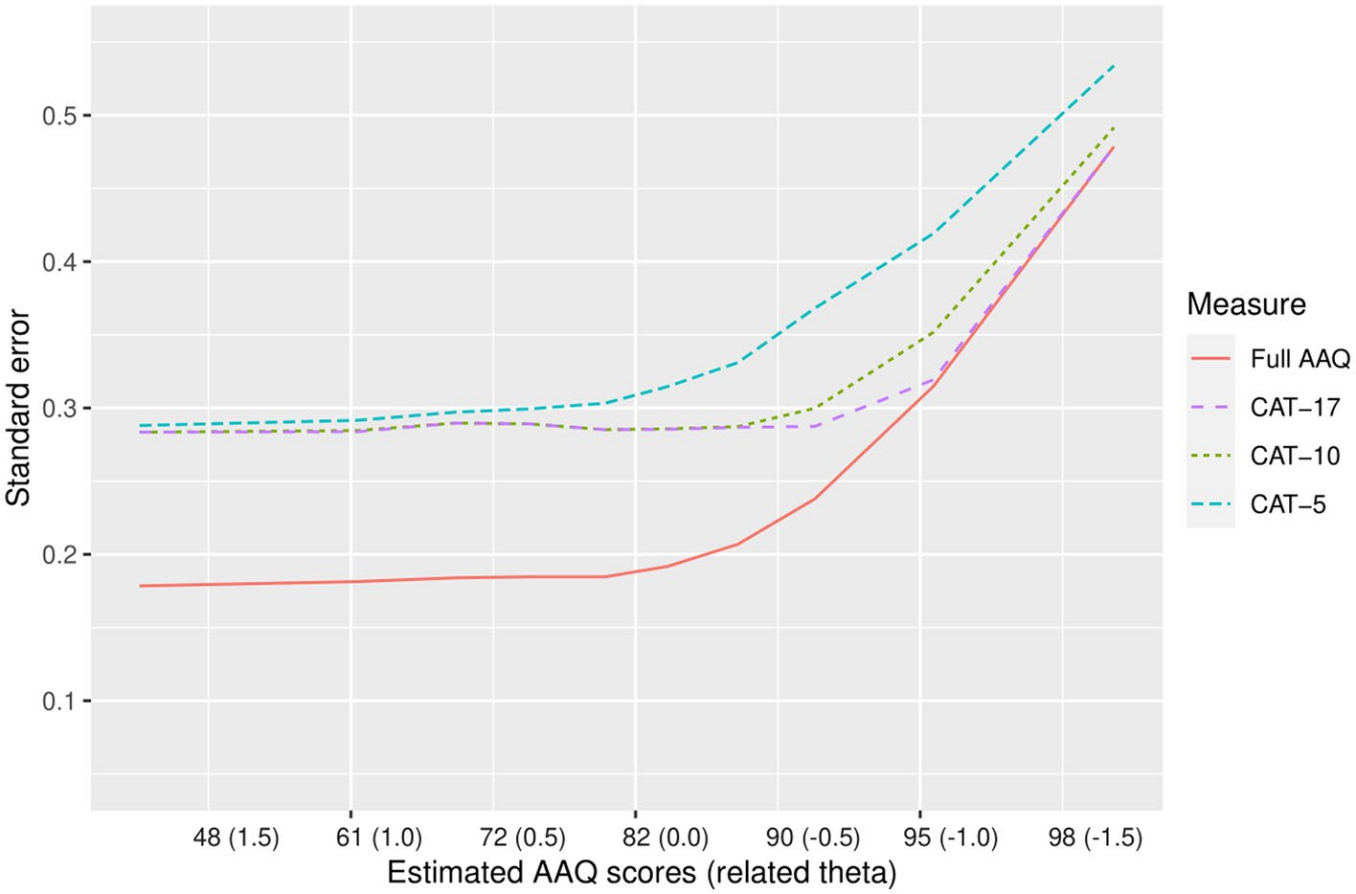
Conditional precision: IRT-based standard error estimates (*y*-axis) for estimated AAQ score and related theta deciles (*x*-axis) in the study sample. Higher AAQ scores indicate less activity limitations

With regard to construct validity, AAQ full measure and CAT scores were highly correlated with PRO (H/KOOS ADL), and PerfO measures (SCT, TUG, and CST), with |*r*| ≥ 0.60. The correlation coefficients between AAQ scores (full measure and CAT versions) and each of the other PRO and PerfO measures tended to be higher than the correlations between the H/KOOS ADL with the SCT (*r* =  − 0.56; 95% CI [− 0.63, − 4.72]), the TUG (*r* =  − 0.48; 95% CI [− 0.56, − 0.40]), and the CST (*r* = 0.48; 95% CI [0.38, 0.56]). The test length of the full AAQ (17 items) was more than halved with each CAT version, with an average of 8.0 items (minimum = 4; maximum = 17) for the CAT without test length restrictions (‘CAT-17’), 6.6 items (minimum = 4; maximum = 10) for the ‘CAT-10’ and 4.8 items (minimum = 4; maximum = 5) for the’CAT-5’. Figure 1 shows that in all CAT versions about 5 to 6 items were sufficient for precise scoring (SE < 0.3) of participants with average and below-average scores (i.e. more severe activity limitation levels). In contrast, for participants with above-average scores (i.e. less severe levels of activity limitations), more items were needed for precise measurement. In accordance with this finding, Fig. 2 shows higher standard errors for patients scoring above-average. When using the ‘CAT-5’, only participants with AAQ scores lower than 80 could be scored with a precision of SE ≤ 0.3. In contrast, using the CAT versions that allow for administering more than 5 items, the range of precise measurement was comparable to the full AAQ measure.

## Discussion

Based on a large international sample of patients with hip and/or knee osteoarthritis, the findings of our study indicate that all items of the AAQ are well-suited for being calibrated on a unidimensional IRT-based scale. Item parameters have been established using graded response modelling. These parameters can be used for applying the AAQ as CAT. Using post-hoc simulations, good psychometric properties were found for three different CAT versions (without length restrictions, with a maximum of 10 items, with a maximum of 5 items).

Statistical analyses indicated that all items of the AAQ fulfill psychometric criteria for IRT modelling. Among others, a core assumption of unidimensional IRT is that all items can be used for the assessment of a common underlying construct (i.e., *activity limitations* in the case of the AAQ). Although the results of a traditional unidimensional CFA were inconsistent, bifactor analysis as well as individual item analyses supported a unidimensional structure of the AAQ items. That the use of bifactor models might be better suited for evaluating sufficient unidimensionality of self-reported data than traditional CFA criteria has been discussed before [37].

Individual item parameters indicated that the AAQ items are generally best suited for measuring more severe activity limitations. Moreover, items 16 (‘putting on shoes’) and 17 (‘taking off shoes’) showed comparably low slopes, indicating low associations of these two items with the underlying construct. In the context of CAT, items with low slopes are generally less informative and, consequently, less likely to be administered by the automated CAT algorithm. Nonetheless, items 16 and 17 appeared to be useful when it comes to scoring individuals with below-average activity limitations and were actually selected by the CAT algorithm for some participants.

Important to consider in relation to CAT administration is that the individual items of the AAQ cover somewhat different aspects of physical activity limitations, i.e., climbing stairs (items 1 and 2), walking (items 3 to 7), rising and sitting down (items 9 to 15), but also activities that require fine motor skills next to hip joint mobility, such as picking up an object from the floor as well as putting on and taking off shoes (items 8, 16, and 17, respectively). Thus, when applied as CAT, content validity of the AAQ might be reduced in case one or more of these aspects are skipped due to the automatized CAT algorithm [39]. Content validity means that a measure represents all aspects of the construct of interest. This issue appeared to be particularly relevant for the 5-item CAT, where as much as five AAQ items were never used for scoring any participant (items 2, 8, 9, 15, and 17). In the 10-item CAT, only one item (item 8) was never used; in the 17-item CAT, all items were used (lowest exposure rate was 17% for item 15). Content balancing has been suggested to be a potential solution when reduced content validity causes systematic bias in CAT assessments, i.e., when the items of a scale appear to measure distinct sub-constructs [23, 39]. Nevertheless, our analyses did not indicate any systematic bias when administering the AAQ as CAT. Each CAT version was highly correlated with the full AAQ and the differences to the original AAQ scores were negligible. Moreover, scores of the full AAQ measure and each CAT version were similarly associated with other PRO and PerfO measures of activity limitations. In sum, based on the results of this study, content balancing seems not to be necessary for any version of the AAQ-CAT. Moreover, the correlation between AAQ scores and each of the other measures of activity limitations tended to be higher than the correlations between PRO and PerfO measures. This finding might empirically reflect the original purpose to develop the AAQ as an innovative assessment tool combining the characteristics of PRO and PerfO measures [15].

This study has some limitations. First, the evaluation of the CAT performance was based on post-hoc simulations. The performance of actual AAQ-CAT administrations must be examined in future studies. Nevertheless, findings of previous studies comparing simulated and real CAT data indicated that results might be similar [49]. Moreover, using post-hoc simulations had the advantage that the anticipated performance of different CAT versions could directly be compared to each other and to the full measure. Second, while 11 language versions of the AAQ already exist, data from only 7 countries were used for psychometric evaluations and for establishing IRT parameters in the present study. Three languages (German, Swedish, and Turkish) could not be considered because sufficient data has not yet been collected. Moreover, Italian data were not considered for establishing CAT parameters in the present study because considerable differential item functioning has been identified before [17]. However, it is not known whether these problems were caused by a lack of cross-cultural validity, or whether there was an issue with the specific set of Italian data collected for the AAQ cross-cultural validity study [17]. As long as cross-cultural invariance has not been shown, CAT results should be interpreted with caution for languages not included in the present study. Third, the current AAQ metric ranging from 0 to 100 is arbitrary. It is yet to be decided whether a different metric should be used. For instance, linearly transforming the IRT-based theta metric to a T-score metric with a mean of 50 and a standard deviation of 10 based on a representative sample of a meaningful reference population might lead to increased interpretability of AAQ scores [33]. Original AAQ scores could also be linked to such a metric, allowing for comparisons with AAQ-CAT scores.

With regard to the comparison of different CAT versions, the CAT-10 (with a maximum of 10 items) appeared to be the most efficient version in our sample, with an average of less than 7 administered items but with comparable precision and validity to the full AAQ. Nevertheless, for samples with highly impaired patients, the CAT-5 might also be well-suited.

The AAQ was originally developed to combine the benefits of patient-reported and performance-based measures of activity limitations. In addition, since the AAQ is almost non-verbal, it is applicable in low literacy patients and its items are easy to translate to other languages, which allows for cross-cultural application. Our study clearly supports the suitability of the AAQ to be applied as CAT, measuring activity limitations with lower respondent burden, but similar precision and construct validity compared to the full AAQ measure. Moreover, calibration of the AAQ to an IRT-based scale is the basis for expanding the measurement range by adding new items, e.g., specific items assessing the extremes of the underlying construct, in future developments. To make the CAT accessible to users, it is considered to integrate the AAQ into existing CAT platforms, e.g., the Dutch-Flemish PROMIS Assessment Center.

## Supplementary Information

Below is the link to the electronic supplementary material.

Supplementary file1 (TIFF 2461 KB)

Supplementary file2 (TIFF 2461 KB)

## Data Availability

Data sharing is not applicable to this article as no new data were created or analyzed in this study.

## References

[1] Pereira D, Peleteiro B, Araújo J, Branco J, Santos RA, Ramos E (2011). The effect of osteoarthritis definition on prevalence and incidence estimates: A systematic review. Osteoarthritis and Cartilage.

[2] Bijlsma JW, Berenbaum F, Lafeber FP (2011). Osteoarthritis: An update with relevance for clinical practice. Lancet.

[3] Smith TO, Mansfield M, Hawker GA, Hunter DJ, March LM, Boers M, Shea BJ, Christensen R, Guillemin F, Terwee CB (2019). Uptake of the OMERACT-OARSI hip and knee osteoarthritis core outcome set: Review of randomized controlled trials from 1997 to 2017. The Journal of Rheumatology.

[4] Walton MK, Powers JH, Hobart J, Patrick D, Marquis P, Vamvakas S, Isaac M, Molsen E, Cano S, Burke LB (2015). Clinical outcome assessments: Conceptual foundation—Report of the ISPOR clinical outcomes assessment–emerging good practices for outcomes research task force. Value in Health.

[5] Latham NK, Mehta V, Nguyen AM, Jette AM, Olarsch S, Papanicolaou D, Chandler J (2008). Performance-based or self-report measures of physical function: Which should be used in clinical trials of hip fracture patients?. Archives of Physical Medicine and Rehabilitation.

[6] Fayers PM, Langston AL, Robertson C (2007). Implicit self-comparisons against others could bias quality of life assessments. Journal of Clinical Epidemiology.

[7] Stratford PW, Kennedy DM (2006). Performance measures were necessary to obtain a complete picture of osteoarthritic patients. Journal of Clinical Epidemiology.

[8] Terwee CB, van der Slikke RM, van Lummel RC, Benink RJ, Meijers WG, de Vet HC (2006). Self-reported physical functioning was more influenced by pain than performance-based physical functioning in knee-osteoarthritis patients. Journal of Clinical Epidemiology.

[9] Liegl G, Obbarius A, Rose M, Fischer KI, Stengel A, Knebel F, Buttgereit F, Nolte S (2022). Frequently used patient-reported outcome (PRO) measures of general physical function were highly correlated with a multi-task performance outcome (PerfO) test battery. Value in Health.

[10] Steultjens MP, Roorda LD, Dekker J, Bijlsma JW (2001). Responsiveness of observational and self-report methods for assessing disability in mobility in patients with osteoarthritis. Arthritis & Rheumatology.

[11] Stratford PW, Kennedy D, Pagura SM, Gollish JD (2003). The relationship between self-report and performance-related measures: Questioning the content validity of timed tests. Arthritis Care & Research.

[12] Stevens-Lapsley JE, Schenkman ML, Dayton MR (2011). Comparison of self-reported knee injury and osteoarthritis outcome score to performance measures in patients after total knee arthroplasty. PM & R: The Journal of Injury, Function, and Rehabilitation.

[13] Coman L, Richardson J (2006). Relationship between self-report and performance measures of function: A systematic review. Canadian Journal on Aging.

[14] Peter WF, Loos M, de Vet HC, Boers M, Harlaar J, Roorda LD, Poolman RW, Scholtes VA, Boogaard J, Buitelaar H, Steultjens M, Roos EM, Guillemin F, Rat AC, Benedetti MG, Escobar A, Østerås N, Terwee CB (2015). Development and preliminary testing of a computerized animated activity questionnaire in patients with hip and knee osteoarthritis. Arthritis Care & Research.

[15] Terwee CB, Coopmans C, Peter WF, Roorda LD, Poolman RW, Scholtes VA, Harlaar J, de Vet HC (2014). Development and validation of the computer-administered animated activity questionnaire to measure physical functioning of patients with hip or knee osteoarthritis. Physical Therapy.

[16] Peter WF, Loos M, van den Hoek J, Terwee CB (2015). Validation of the animated activity questionnaire (AAQ) for patients with hip and knee osteoarthritis: Comparison to home-recorded videos. Rheumatology International.

[17] Peter WF, de Vet HCW, Boers M, Harlaar J, Roorda LD, Poolman RW, Scholtes VAB, Steultjens M, Hendry GJ, Roos EM, Guillemin F, Benedetti MG, Cavazzuti L, Escobar A, Dagfinrud H, Terwee CB (2017). Cross-cultural and construct validity of the animated activity questionnaire. Arthritis Care & Research.

[18] Peter WF, Dagfinrud HS, Østerås N, Terwee CB (2017). Animated activity questionnaire (AAQ), a new method of self-reporting activity limitations in patients with hip and knee osteoarthritis: Comparisons with observation by spouses for construct validity. Musculoskeletal Care.

[19] Peter WF, Poolman RW, Scholtes VAB, de Vet HCW, Terwee CB (2019). Responsiveness and interpretability of the animated activity questionnaire for assessing activity limitations of patients with hip or knee osteoarthritis. Musculoskeletal Care.

[20] Kean, J., & Reilly, J. (2014). Item response theory. In *Handbook for clinical research: Design, statistics and implementation* (pp. 195–198).

[21] Cella D, Gershon R, Lai J-S, Choi S (2007). The future of outcomes measurement: Item banking, tailored short-forms, and computerized adaptive assessment. Quality of Life Research.

[22] Fries JF, Witter J, Rose M, Cella D, Khanna D, Morgan-DeWitt E (2014). Item response theory, computerized adaptive testing, and PROMIS: Assessment of physical function. The Journal of Rheumatology.

[23] Bjorner JB, Chang C-H, Thissen D, Reeve BB (2007). Developing tailored instruments: Item banking and computerized adaptive assessment. Quality of Life Research.

[24] Petersen MA, Aaronson NK, Arraras JI, Chie W-C, Conroy T, Costantini A, Dirven L, Fayers P, Gamper E-M, Giesinger JM (2018). The EORTC CAT Core—The computer adaptive version of the EORTC QLQ-C30 questionnaire. European Journal of Cancer.

[25] De Groot I, Reijman M, Terwee C, Bierma-Zeinstra S, Favejee M, Roos E, Verhaar J (2007). Validation of the Dutch version of the hip disability and osteoarthritis outcome score. Osteoarthritis and Cartilage.

[26] De Groot IB, Favejee MM, Reijman M, Verhaar JA, Terwee CB (2008). The Dutch version of the knee injury and osteoarthritis outcome score: A validation study. Health and Quality of Life Outcomes.

[27] Rejeski WJ, Ettinger WH, Schumaker S, James P, Burns R, Elam JT (1995). Assessing performance-related disability in patients with knee osteoarthritis. Osteoarthritis and Cartilage.

[28] Steffen TM, Hacker TA, Mollinger L (2002). Age-and gender-related test performance in community-dwelling elderly people: Six-Minute walk test, berg balance scale, timed up & go test, and gait speeds. Physical Therapy.

[29] Jones CJ, Rikli RE, Beam WC (1999). A 30-s chair-stand test as a measure of lower body strength in community-residing older adults. Research Quarterly for Exercise and Sport.

[30] Dobson F, Hinman RS, Roos EM, Abbott JH, Stratford P, Davis AM, Buchbinder R, Snyder-Mackler L, Henrotin Y, Thumboo J (2013). OARSI recommended performance-based tests to assess physical function in people diagnosed with hip or knee osteoarthritis. Osteoarthritis and Cartilage.

[31] de Nascimento CD, Peter WF, Ribeiro IM, de Souza Moreira B, Lima VP, de Carvalho Bastone A (2021). Cross-cultural validity of the animated activity questionnaire for patients with hip and knee osteoarthritis: A comparison between the Netherlands and Brazil. Brazilian Journal of Physical Therapy.

[32] Altman R, Alarcon G, Appelrouth D, Bloch D, Borenstein D, Brandt K, Brown C, Cooke T, Daniel W, Feldman D (1991). The American College of Rheumatology criteria for the classification and reporting of osteoarthritis of the hip. Arthritis & Rheumatism.

[33] Reeve BB, Hays RD, Bjorner JB, Cook KF, Crane PK, Teresi JA (2007). Psychometric evaluation and calibration of health-related quality of life item banks: Plans for the patient-reported outcomes measurement information system (PROMIS). Medical Care.

[34] Nguyen TH, Han H-R, Kim MT, Chan KS (2014). An introduction to item response theory for patient-reported outcome measurement. Patient.

[35] Rosseel Y (2012). lavaan: An R package for structural equation modeling. Journal of Statistical Software.

[36] Reise SP, Scheines R, Widaman KF, Haviland MG (2013). Multidimensionality and structural coefficient bias in structural equation modeling: A bifactor perspective. Educational and Psychological Measurement.

[37] Cook KF, Kallen MA, Amtmann D (2009). Having a fit: Impact of number of items and distribution of data on traditional criteria for assessing IRT’s unidimensionality assumption. Quality of Life Research.

[38] Stochl J, Jones PB, Croudace TJ (2012). Mokken scale analysis of mental health and well-being questionnaire item responses: A non-parametric IRT method in empirical research for applied health researchers. BMC Medical Research Methodology.

[39] Liegl G, Rose M, Knebel F, Stengel A, Buttgereit F, Obbarius A, Fischer HF, Nolte S (2020). Using subdomain-specific item sets affected PROMIS physical function scores differently in cardiology and rheumatology patients. Journal of Clinical Epidemiology.

[40] Samejima F, van der Linden W, Hambleton R (1997). Graded response model. Handbook of modern item response theory.

[41] Haberman SJ, Sinharay S, Chon KH (2013). Assessing item fit for unidimensional item response theory models using residuals from estimated item response functions. Psychometrika.

[42] Xu C, Schaverien MV, Christensen JM, Sidey-Gibbons CJ (2022). Efficient and precise Ultra-QuickDASH scale measuring lymphedema impact developed using computerized adaptive testing. Quality of Life Research.

[43] Magis D, Raîche G (2012). Random generation of response patterns under computerized adaptive testing with the R package catR. Journal of Statistical Software.

[44] Chalmers RP (2012). mirt: A multidimensional item response theory package for the R environment. Journal of Statistical Software.

[45] R Core Team (2014). R: A language and environment for statistical computing.

[46] Revelle W (2016). Psych: Procedures for personality and psychological research.

[47] van der Ark LA (2007). Mokken scale analysis in R. Journal of Statistical Software.

[48] Choi SW, Gibbons LE, Crane PK (2011). Lordif: An R package for detecting differential item functioning using iterative hybrid ordinal logistic regression/item response theory and Monte Carlo simulations. Journal of Statistical Software.

[49] Kocalevent R-D, Rose M, Becker J, Walter OB, Fliege H, Bjorner JB, Kleiber D, Klapp BF (2009). An evaluation of patient-reported outcomes found computerized adaptive testing was efficient in assessing stress perception. Journal of Clinical Epidemiology.

