# Correction to: Suitability of the animated activity questionnaire for use as computer adaptive test: establishing the AAQ‑CAT

**DOI:** 10.1007/s11136-023-03490-2

**Published:** 2023-07-26

**Authors:** Gregor Liegl, Leo D. Roorda, Caroline B. Terwee, Martijn Steultjens, Ewa M. Roos, Francis Guillemin, Maria Grazia Benedetti, Hanne Dagfnrud, Alessandra de Carvalho Bastone, Wilfred F. Peter

**Affiliations:** 1 Center for Patient-Centered Outcomes Research, Charité– Universitätsmedizin Berlin, corporate member of Freie Universität Berlin and Humboldt-Universität Zu Berlin, Berlin, Germany; 2 Amsterdam Rehabilitation Research Center | Reade, Amsterdam, The Netherlands; 3 Department of Epidemiology and Data Science, Amsterdam UMC, Vrije Universiteit Amsterdam, Amsterdam, The Netherlands; 4 Amsterdam Public Health Research Institute, Amsterdam, The Netherlands; 5 School of Health and Life Sciences, Glasgow Caledonian University, Glasgow, UK; 6 Institute of Sports Science and Clinical Biomechanics, University of Southern Denmark, Odense, Denmark; 7 EA 4360 APEMAC, Inserm CIC-EC 1433, University Hospital, Université de Lorraine, Nancy, France; 8 Physical Medicine and Rehabilitation Unit, IRCCS Istituto Ortopedico Rizzoli, Bologna, Italy; 9 Diakonhjemmet Hospital, Oslo, Norway; 10 University of Oslo, Oslo, Norway; 11 Department of Physical Therapy, Universidade Federal dos Vales do Jequitinhonha e Mucuri (UFVJM), Diamantina, MG Brazil

**Correction to: Quality of Life Research (2023) 32:2403–2413** 10.1007/s11136-023-03402-4

The authors regret that the affiliation of Professor Maria Grazia Benedetti published in the article was not correct. Hence, the updated affiliation of the author is provided in this correction.

The authors would like to apologise for any inconvenience caused.

