# Supplementary figures and images for: Suitability of the animated activity questionnaire for use as computer adaptive test: establishing the AAQ-CAT
Source: Qual Life Res. 2023 Apr 3;32(8):2403–13. doi: 10.1007/s11136-023-03402-4 (PMC10329068; doi:10.1007/s11136-023-03402-4)

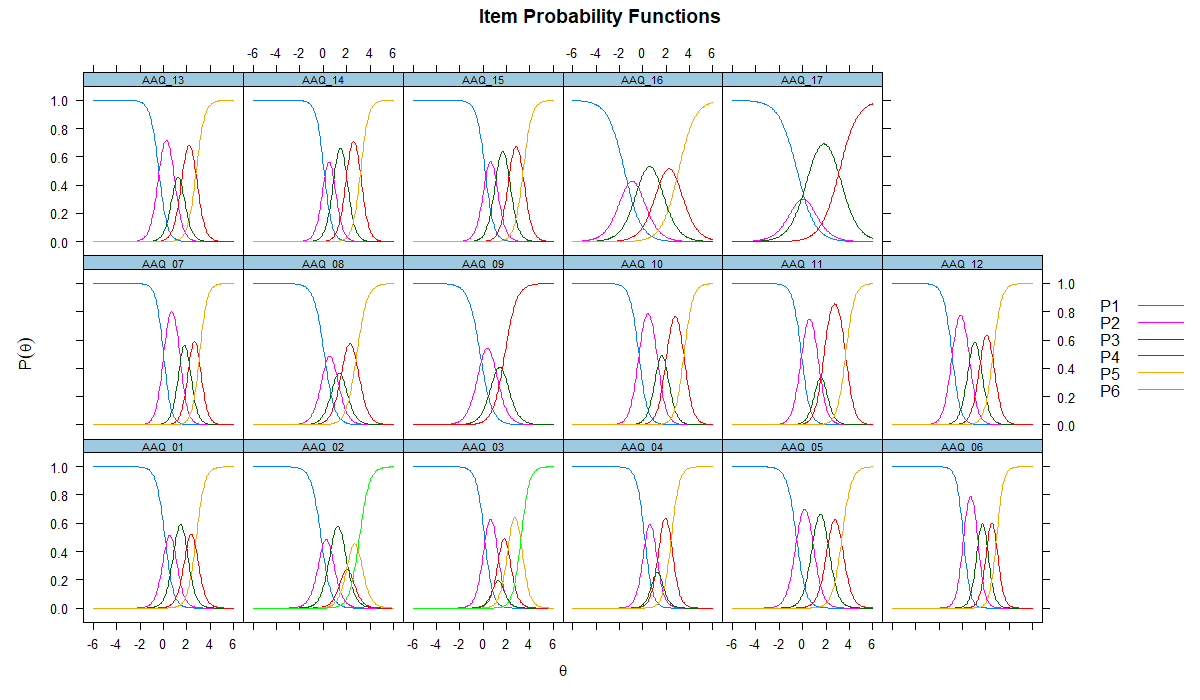

Supplement: Supplementary file 1 — Supplementary file1 (TIFF 2461 KB) [file 11136_2023_3402_MOESM1_ESM.tiff]

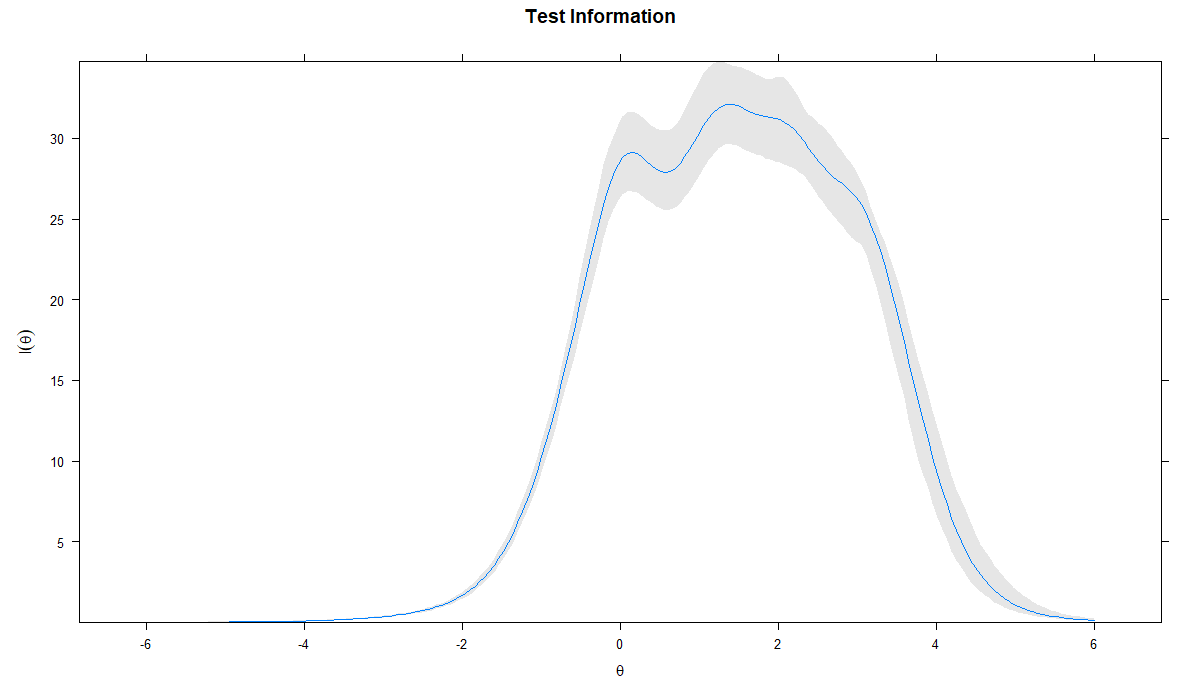

Supplement: Supplementary file 2 — Supplementary file2 (TIFF 2461 KB) [file 11136_2023_3402_MOESM2_ESM.tiff]
